# Asymptomatic and symptomatic recurrence of TB among cured pulmonary TB patients

**DOI:** 10.5588/ijtldopen.26.0210

**Published:** 2026-10-12

**Authors:** M. Paradkar, S. Gaikwad, C. Padmapriyadarsini, M. Naik, N. Pradhan, R. Karyakarte, T. Sahasrabudhe, R. Lokhande, S. Deshmukh, M. Barthwal, S. Raskar, K. Thiruvengadam, A. Kakrani, S. Krishnan, A. Gupte, A. Gupta, N. Gupte, J.E. Golub, E.A. Kendall, V. Mave, on behalf of the TBDM-CTRIUMPh-RePORT India Study Team

**Affiliations:** 1 BJ Government Medical College-Johns Hopkins Clinical Research Site, Pune, India;; 2 Johns Hopkins Center for Infectious Diseases in India, Pune, India;; 3 BJ Government Medical College and Sassoon General Hospitals, Pune, India;; 4 Indian Council of Medical Research, National Institute for Research in Tuberculosis, Chennai, India;; 5 Dr. D.Y. Patil Medical College, Hospital & Research Centre, Pune, India;; 6 Johns Hopkins University School of Medicine, Baltimore, MD, USA;; 7 Boston University School of Public Health, Boston, MA, USA.

**Keywords:** tuberculosis, India, asymptomatic recurrence, symptomatic recurrence

## Abstract

**BACKGROUND:**

Asymptomatic pulmonary TB (PTB), including asymptomatic recurrence, has emerged as a potential contributor to TB transmission.

**METHODS:**

Participants ≥14 years old from two Indian cohorts with culture-confirmed, drug-sensitive PTB and an outcome of cure were followed up for up to 18 months post treatment completion. We calculated the cumulative incidence and timing of symptomatic TB recurrence and the prevalence of asymptomatic recurrence at two screening time points, and performed Cox-regression analysis.

**RESULTS:**

Of 713 culture-confirmed PTB and cure, 60 (8%) experienced confirmed TB recurrence (cumulative incidence: 8.7 [95% confidence interval (CI): 6.6–11.2]/100 person-years), including 40 (66.7%) symptomatic and 20 (33.3%) asymptomatic recurrences. At study visits with scheduled sputum testing, 22 of 35 (63%) TB recurrences diagnosed were asymptomatic at the time of diagnosis. Undernutrition was an independent risk factor for overall TB recurrence (adjusted hazard ratio [aHR] 2.1, 95% CI: 1.1–4.0, *P* = 0.03). Symptomatic recurrences were associated with age > 35 years (aHR 2.5, 95% CI: 1.3–5.0, *P* = 0.01), while the risk of asymptomatic recurrences was higher in the age-group of 14–35 years (aHR 2.8, 95% CI: 0.8–9.8, *P* = 0.10).

**CONCLUSIONS:**

Among cured PTB patients, 8.4% experienced recurrent TB and one third of recurrences were asymptomatic at diagnosis, highlighting a public health concern due to substantial hidden burden of recurrent TB disease in India.

Despite global efforts towards optimising prevention, early detection, and prompt treatment initiation for TB,^1,2^ the rates of decline in TB incidence and mortality are insufficient to achieve the WHO target to end the TB epidemic by 2030.^1–3^ An estimated 10.7 million people developed TB and 1.23 million died of TB worldwide in 2024.^2^ Among several reasons for this persistent disease burden, asymptomatic TB may be a key factor in propagating *Mycobacterium tuberculosis* (*Mtb*) transmission, undermining TB control strategies.^4,5^ Both national population-based surveys and household contact investigations detect *Mtb* bacilli in sputum of asymptomatic individuals.^4–10^ A recent individual participant data meta-analysis of national prevalence surveys showed the proportion of asymptomatic TB to be varying between 40% and 60% among bacteriologically confirmed TB cases depending on the case definitions used, and a quarter of those without cough have positive sputum smears, suggesting infectiousness.^5^ However, evidence on asymptomatic TB pathogenesis, incidence, and risk factors is scarce.^11–13^

An important contributor to the burden of infectious TB is recurrence (including TB relapses and TB reinfections).^14–20^ In India’s national prevalence survey in 2019–2021, 27% of the TB cases detected had recurrent TB (prevalence: 118 cases per 100,000 population), and the proportion of asymptomatic recurrent TB was 41%.^21^ More recently, Naidoo et al. described the incidence of recurrent asymptomatic TB among people living with HIV (PLWH) in South Africa.^22^ But that study enrolled PLWH only after completion of their initial TB treatment, limiting the assessment of treatment-related risk factors. However, the risk of asymptomatic TB recurrence is not described among HIV-negative population comprising majority of people with TB globally and in India, the country with the largest absolute TB burden.^23^

We therefore utilised two longitudinal TB cohort studies in India to assess the cumulative incidence, the risk factors, and the timing of asymptomatic and symptomatic recurrent TB diagnoses, among pulmonary TB (PTB) participants, following TB cure.^24,25^

## METHODS

Individuals with active PTB were recruited to two observational cohorts, namely ‘The Cohort for TB Research by the Indo-US Medical Partnership (CTRIUMPh)’ and ‘Impact of Diabetes on TB Treatment Outcomes (TBDM)’. These cohort studies were conducted at Byramjee Jeejeebhoy Government Medical College and Dr D.Y. Patil University, Pune; and National Institute of Research in Tuberculosis, Chennai, India; in collaboration with Johns Hopkins University, Baltimore, USA; between 2013 and 2019.^24,25^

### Study procedures

The study procedures were similar in both CTRIUMPh and TBDM cohorts. Participants had been diagnosed with new PTB without known drug resistance, with or with microbiological confirmation (Xpert MTB/RIF, acid-fast bacilli [AFB] microscopy or culture), and were enrolled within 14 days of anti-TB treatment (ATT) initiation. The TBDM cohort excluded PLWH. All participants received 6 months of ATT, with thrice-weekly therapy before 2017 and daily regimens after 2017. Participants underwent regular scheduled follow-up visits while on treatment, and during the post-treatment period both cohorts followed up participants every 6 months. Post-treatment follow-up lasted for approximately 12 months in the TBDM cohort (with scheduled study visits at 6 and 12 months after the end of treatment, respectively), and for approximately 18 months in the CTRIUMPh cohort (with scheduled study visits at 6, 12, and 18 months after the end of treatment, respectively). Additionally, an unscheduled visit was conducted whenever a participant presented with suspected TB recurrence (symptoms or signs suggestive of a TB recurrence) or reported that an external clinical care provider had diagnosed the recurrent TB. When a participant missed a scheduled visit window but was able to visit the study clinic at a later time point, an unscheduled visit was performed to conduct the missed evaluations. If the participant was unable to visit at a scheduled or unscheduled time point, then the visit was considered to be missed. At the baseline and at all follow-up visits, socio-demographic, clinical, and laboratory data were collected, including a standard TB symptom screening questionnaire that asked about cough, fever, night sweats, weight loss, swollen glands, and any other symptoms. Chest imaging by X-ray was done at the baseline (in both studies) and week 24 visit (in CTRIUMPh study). At baseline, Xpert MTB/RIF (Cepheid, Sunnyvale, CA, USA), Ziehl–Neelsen smear microscopy for AFB (AFB smear), and cultures by liquid Mycobacterium Growth Incubator Tube (MGIT) and solid Löwenstein–Jensen (LJ) culture media were performed. Prior to the end of ATT, AFB smear and cultures (MGIT and LJ) were performed at all study visits. After the end of ATT, microbiologic testing (AFB smear, MGIT, and LJ) was performed at the following visits (or at the corresponding unscheduled visit for participants who missed the defined visit window period): at 12-month visit in TBDM, at 18-month visit in both TBDM and CTRIUMPh, and any time an unscheduled visit was triggered by symptoms and suspected recurrence in either study. The maximum plasma concentration of each drug was determined at month 1 of ATT using blood samples drawn 2 h post-dose, in a subset that had pharmacokinetic data available. Subtherapeutic cut-offs were defined as rifampicin <8 µg/mL, isoniazid <3 µg/mL, and pyrazinamide <20 µg/mL.

### Outcomes and definitions

The primary analysis was conducted among adult and adolescent participants (14 years of age or older) with newly diagnosed, culture-confirmed PTB at study entry, who had completed at least 6 months of ATT and were declared to be cured of TB. TB cure was defined as clinical response (absence of any TB symptoms or signs at the completion of initial ATT), and a negative AFB smear and culture at the end of treatment. The study outcomes were cumulative incidence and the risk factors, for recurrent TB, stratified by asymptomatic versus symptomatic status at the time of diagnosis. Asymptomatic recurrent TB was defined as detection of *Mtb* bacilli by sputum culture, in absence of any reported TB symptoms (including any duration of cough, fever, night sweats, or weight loss) at the time of microbiological positivity. Symptomatic recurrent TB was defined as a recommendation for TB retreatment in the presence of TB symptoms of any duration (cough, fever, night sweats, weight loss, or any other clinical symptoms or signs or radiological findings suggestive of either pulmonary and/or extra-pulmonary TB), with a positive TB culture. Undernutrition was defined as a body mass index (BMI) < 18.5 kg/m^2^.

We evaluated the timing of asymptomatic and symptomatic recurrent TB diagnoses relative to the timing of scheduled microbiological testing at study visits post ATT completion. For each scheduled post-treatment visit that included microbiological testing, we calculated the proportion of diagnoses at that visit that were asymptomatic; and examined when the participant had last undergone negative microbiological testing to place an upper bound on the potential duration of asymptomatic TB in these individuals.

### Statistical methods

TB recurrence rates were calculated per 100 person-years (PY) of follow-up. Participants lost to follow-up were censored for the remaining duration of follow-up. We used Cox-proportional-hazard regression to evaluate the association of risk factors with recurrent TB outcomes. The participants diagnosed with asymptomatic recurrent TB were excluded from the symptomatic recurrent TB analysis, and vice versa, to avoid assignment bias.

In addition, we did a sensitivity analysis that also included those without culture evidence of cure; that is, we included those with merely a clinical response to initial treatment regardless of whether an end-of-treatment culture was performed.

### Ethical statement

The CTRIUMPh and TBDM studies were approved by the Institutional Ethics Committees or the Institutional Review Board at each of the participating sites. All the participants or legal guardians aged 18 years and above provided written informed consent, and assent was obtained from the participants between 8 and 18 years of age.

## RESULTS

Of 1,196 patients at least 14 years old with newly diagnosed, drug-sensitive PTB enrolled, 713 (60%) met inclusion criteria for the primary analysis (culture-confirmed TB at entry followed by culture-confirmed TB cure) – see Figure 1. Of these, 452 (63%) were male, 323 (45%) > 35 years of age, 405 (57%) undernourished, 229 (32%) had diabetes mellitus, 8 (1%) PLWH, 128 (18%) reported smoking, 223 (31%) consumed alcohol, and 587 (82%) had received a thrice-weekly ATT regimen (Table). During a median 10.9 months (interquartile range [IQR]: 9.8–16.5) of follow-up after treatment completion, 60 (8%) participants developed culture-confirmed TB recurrence, at a cumulative incidence rate of 8.7 (95% confidence interval [CI]: 6.6–11.2)/100 PY.

**Figure 1.**
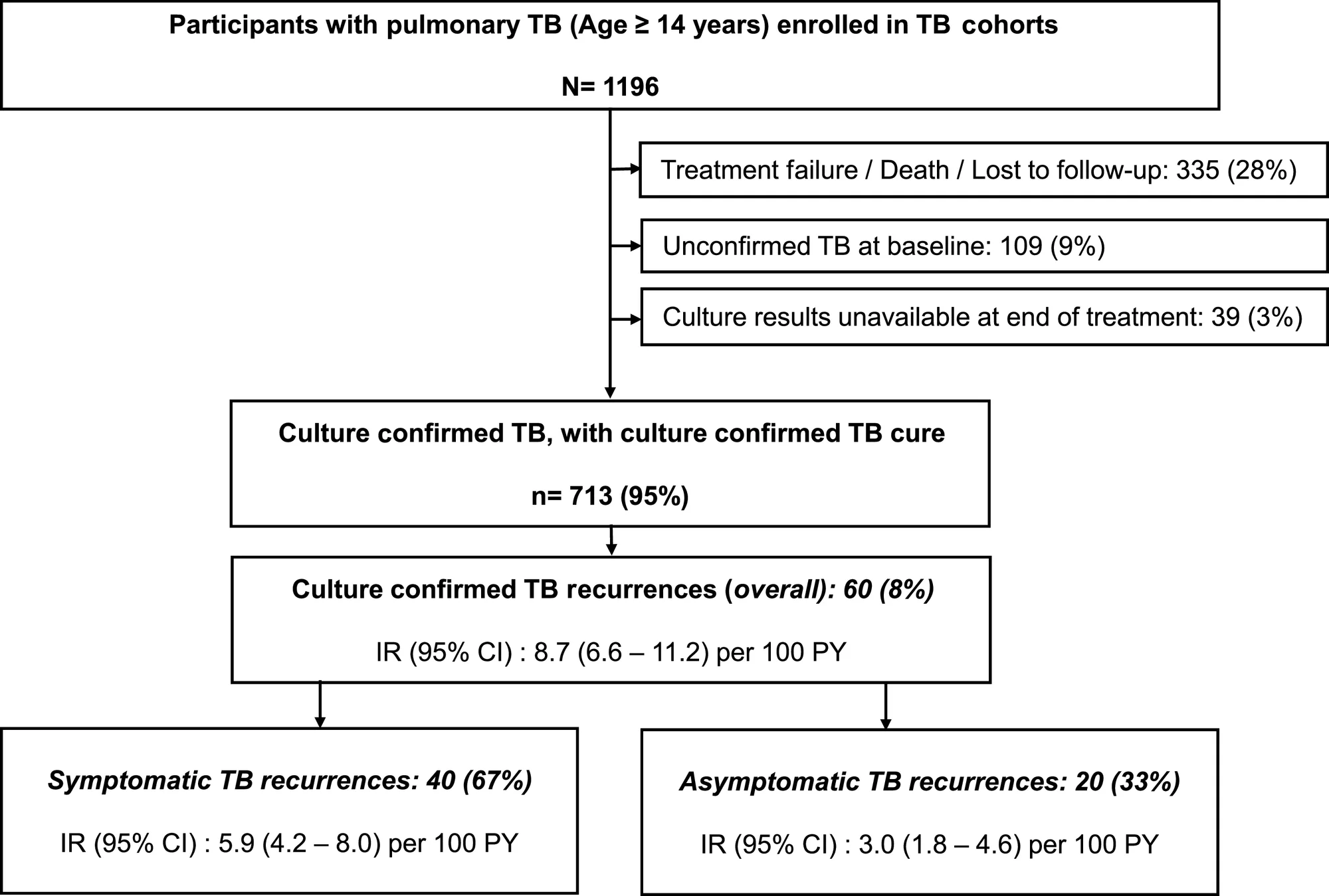
Flow chart depicting recruitment of pulmonary TB cases in the study cohorts, and cumulative incidence rates of culture-confirmed TB recurrence (overall, asymptomatic, and symptomatic), among TB cases with culture-confirmed TB cure in India. CI = confidence interval; IR = incidence rate; PY = person-years.

**Table.** Characteristics of adult confirmed pulmonary TB cases with satisfactory clinical response to treatment, in Indian cohorts.

	Culture-confirmed TB with culture-confirmed cure (N = 713)		Culture-confirmed TB with clinical response, with or without culture-confirmed cure (N = 752)	
Characteristics	N	%	n	%
Gender				
Men	452	63	478	64
Women	261	37	274	36
Age (years)				
> 35	323	45	340	45
14–35	390	55	412	55
Body mass index				
Normal	269	38	282	38
Obese	38	6	42	6
Underweight	405	57	427	57
HIV infection				
No	701	98	739	98
Yes	8	1	9	1
Diabetes mellitus				
No	484	68	505	67
Yes	229	32	247	33
Ever smoking				
No	519	73	544	72
Yes	128	18	141	19
Alcohol consumption				
No	463	65	488	65
Yes	223	31	236	31
Treatment regimen				
Thrice weekly	587	82	618	82
Daily	126	18	134	18
Baseline AFB smear				
Negative	123	17	133	18
Positive	590	83	619	82
Month 2 AFB smear				
Negative	514	72	538	72
Positive	56	8	59	8
Month 2 culture				
Negative	541	76	558	74
Positive	109	15	114	15

AFB = acid-fast bacilli.

### Timing and modes of recurrent TB diagnosis

Of these 60 TB recurrences, 40 (67%) were symptomatic at the time of diagnosis, and 20 (33%) were asymptomatic (Figure 2). The corresponding cumulative incidence rates were 5.9 (4.2–8.0) and 3.0 (IQR 1.8–4.6) per 100 PY for symptomatic and asymptomatic TB diagnoses, respectively. The median time to diagnosis for all recurrences was 5.8 months (IQR 4.7–9.6) post ATT completion. Median time to diagnosis of symptomatic TB recurrence was 5.4 months (IQR 4.5–8.0) post ATT completion. Of the 40 symptomatic recurrent TB cases, 13 (33%) were diagnosed at scheduled study visits, 23 (58%) at unscheduled (symptom-triggered) study visits, and 4 (10%) by non-research clinicians. Among those diagnosed with symptomatic recurrence at study visits, the median reported duration of symptoms was only 18 days (IQR 10–45). Only 12 (30%) of those diagnosed with symptomatic recurrent TB had undergone mycobacteriological testing since their end-of-treatment study visit; this testing had occurred median 5.3 months (IQR 4.0–5.9) prior to diagnosis, and only one patient had completed negative microbiological testing within the preceding 3 months (Figure 2).

**Figure 2.**
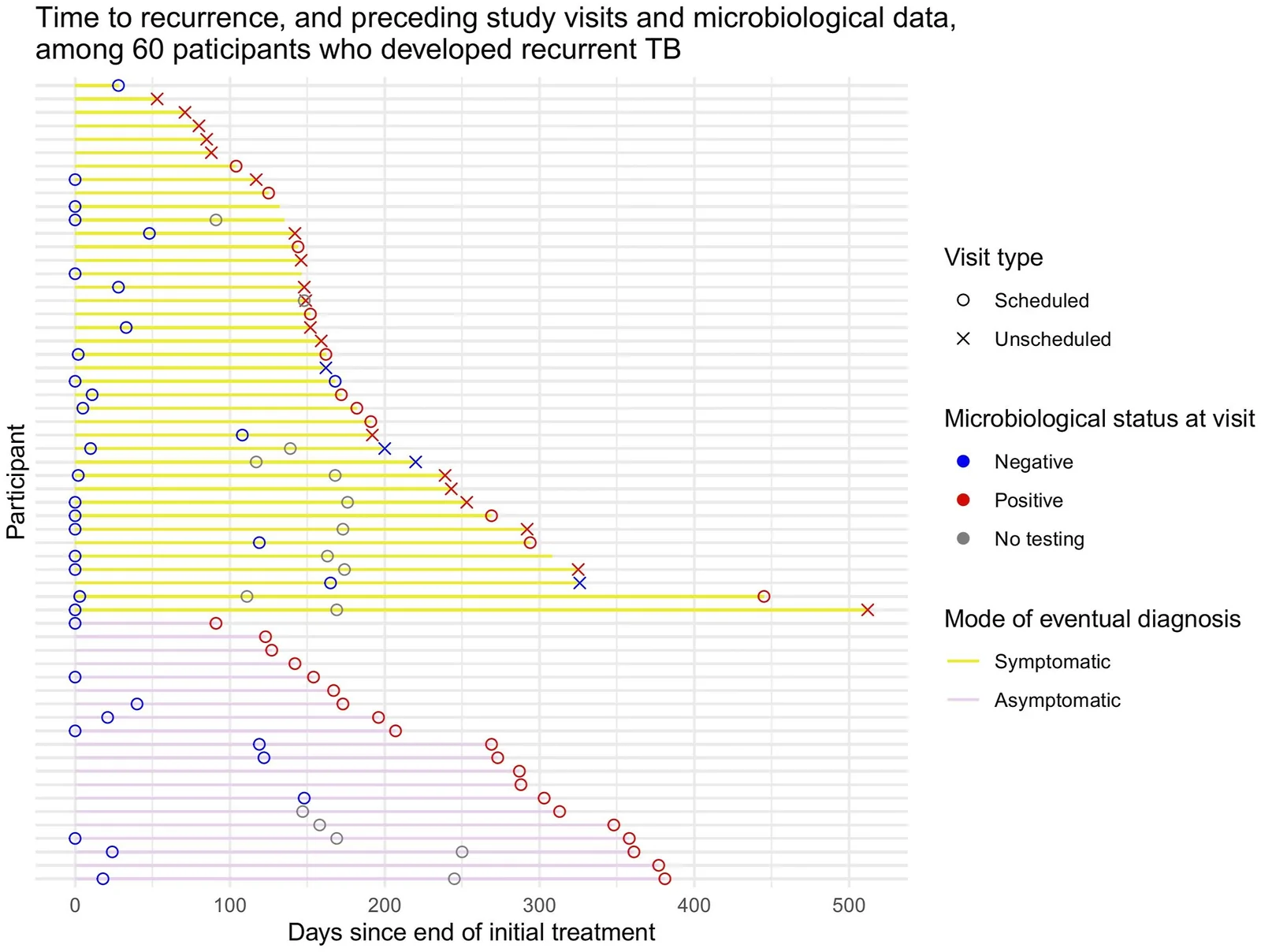
Timing of mode of recurrent TB diagnosis and preceding study visits, among TB cases with culture-confirmed TB cure in India. Each horizontal line represents the duration of post-treatment follow-up for one patient who was eventually diagnosed with recurrent symptomatic (lavender lines) or asymptomatic (yellow lines) TB. Study visits that occurred at or after the end of treatment are represented as shapes and are colour-coded by microbiological status (smear and/or culture; positive if either had a positive result) at that visit.

Asymptomatic TB recurrence was detected a median 9.0 months (IQR 5.5–10.7) post ATT completion. Of the TB diagnosed at the 18-month visit (when nearly all participants underwent microbiological testing), 11 of 12 cases were asymptomatic; similarly, of diagnoses made at the 12-month visit in the TBDM study, 10 of 17 were asymptomatic. Asymptomatic TB was detected more frequently by the TBDM study (3.3%), which conducted microbiological testing at both the 12- and the 18-month visits, than by the CTRIUMPh study (2.2%), which performed microbiological testing only at 18 months (Supplementary Data Table S1).

### Risk factors for TB recurrence (n = 713)

In the univariable analysis, male gender (unadjusted hazard ratio [uHR] 1.7, 95% CI: 1.0–3.3, *P* = 0.07) and undernutrition (uHR 2.0, 95% CI: 1.1–3.7, *P* = 0.02) were associated with overall TB recurrence. In a model adjusted for age, gender, and month 2 culture results, undernutrition (adjusted hazard ratio [aHR] 2.1, 95% CI: 1.1–4.0, *P* = 0.03) was found to be an independent risk factor for TB recurrence (Figure 3; Supplementary Data Table S2). In the analysis limited to recurrences that were symptomatic at the time of diagnosis, age > 35 years (aHR 2.5, 95% CI: 1.3–5.0, *P* = 0.01) and undernutrition (aHR 2.2, 95% CI: 1.0–4.7, *P* = 0.04) were independent risk factors for symptomatic TB recurrence after adjusting for gender (Supplementary Data Figure S1 and Table S3). When limiting analysis to recurrences that were diagnosed at the asymptomatic stage, multivariable model adjusted for age, gender, BMI, and month 2 culture results showed that age-group 14–35 years (aHR 2.8, 95% CI: 0.8–9.8, *P* = 0.10), male gender (aHR 2.0, 95% CI: 0.67–10.0, *P* = 0.21), undernutrition (aHR 2.0, 95% CI: 0.5–7.3, *P* = 0.3) at baseline, and positive month 2 culture (aHR 2.3, 95% CI: 0.8–6.8, *P* = 0.13) had estimated HRs of at least 2, although the sample size was small (N = 20) and no risk factors reached statistical significance (Figure 4; Supplementary Data Table S4).

**Figure 3.**
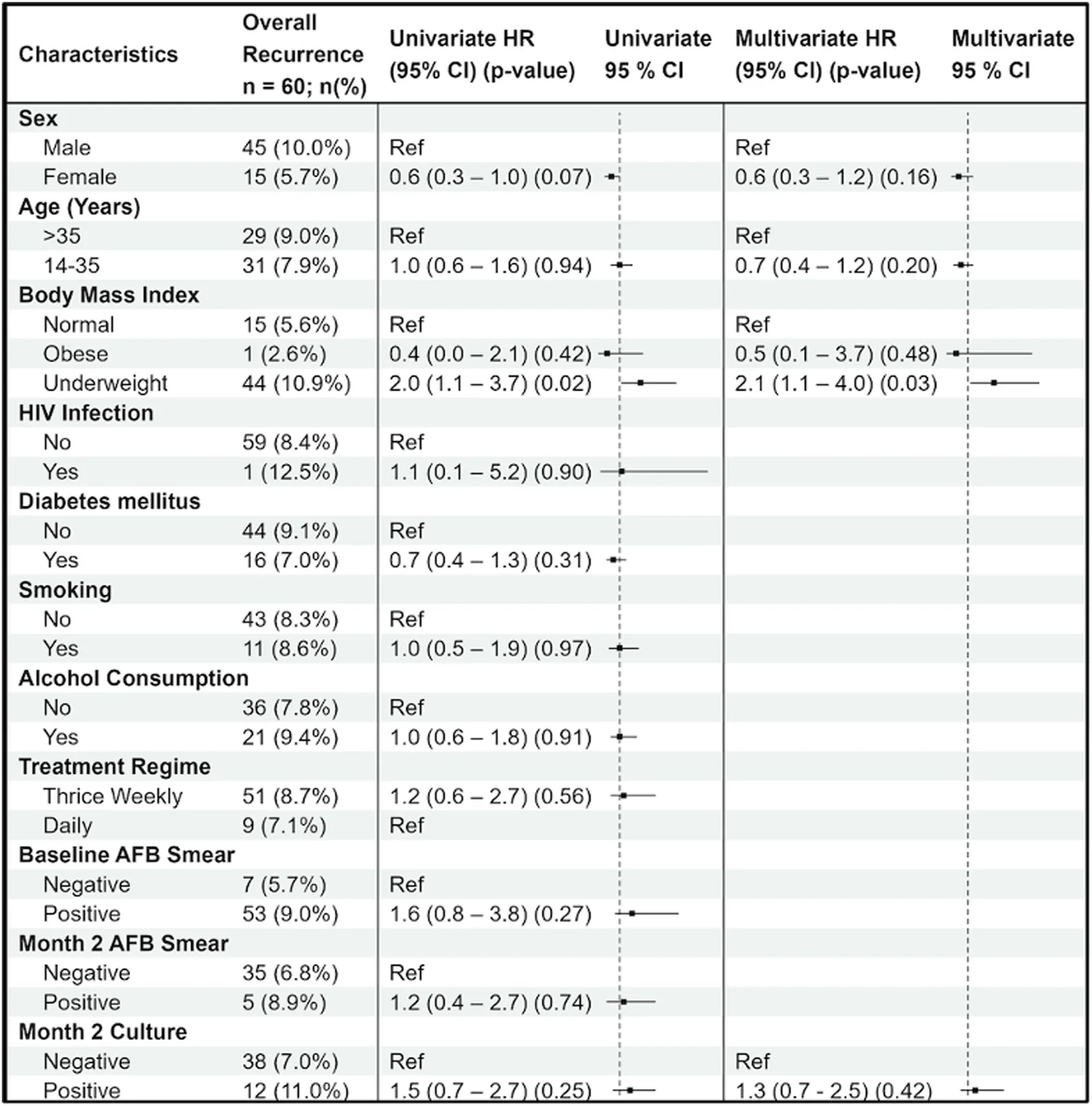
Forest plot showing risk factors for culture-confirmed TB recurrence (overall), among culture-confirmed pulmonary TB cases with culture-confirmed TB cure. HR = hazard ratio; CI = confidence interval; AFB = acid-fast bacilli; Ref = reference.

**Figure 4.**
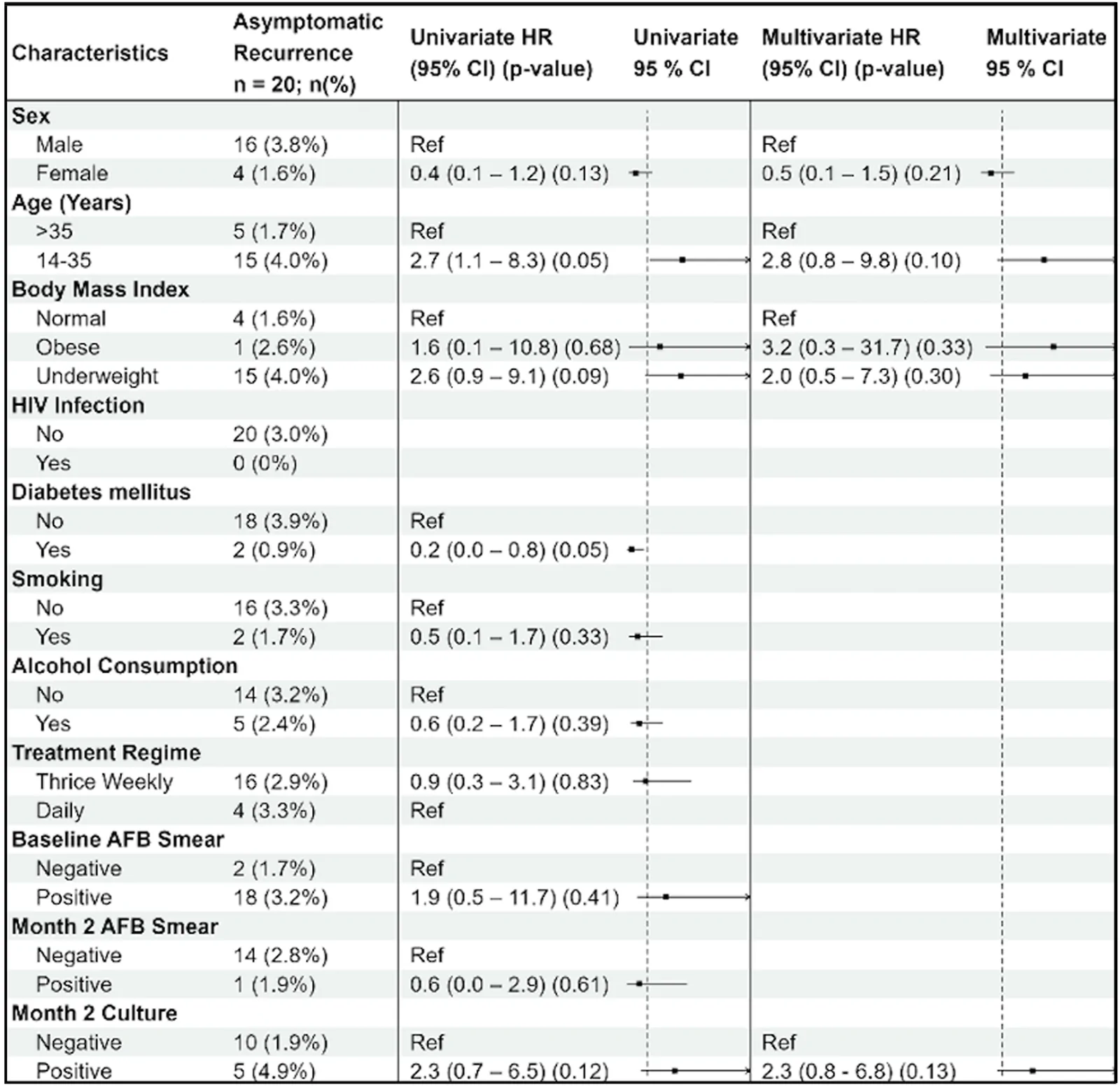
Forest plot showing risk factors for culture-confirmed asymptomatic TB recurrence among culture-confirmed pulmonary TB cases with culture-confirmed TB cure. HR = hazard ratio; CI = confidence interval; AFB = acid-fast bacilli; Ref = reference.

### TB recurrence and pharmacokinetic drug concentrations

Of the 713 participants, subsets of 338 (49%), 353 (50%), and 338 (47%) underwent drug concentration testing for rifampicin, isoniazid, and pyrazinamide, respectively. Among those without recurrence, we observed 267 (89%), 65 (20%), and 56 (17%) had subtherapeutic concentrations of rifampicin, isoniazid, and pyrazinamide, respectively. Among those with recurrence, we observed 24 (89%), 5 (19%), and 6 (24%) had subtherapeutic concentrations of rifampicin, isoniazid, and pyrazinamide, respectively. In the univariate analysis, subtherapeutic pyrazinamide concentrations (uHR 1.6, 95% CI 0.6–3.9, *P* = 0.33) showed a 1.6-fold higher risk of TB recurrence. However, this finding was not statistically significant in our analysis (Supplementary Data Tables S5 and S6).

### Sensitivity analysis

We replicated the primary analysis among the extended cohort of all participants with clinical response to TB treatment, including those with and without culture evidence of TB cure (n = 752, TB recurrences = 65) – see Supplementary Data Tables S7–S9. The results were consistent with that of the primary analysis. Specifically, undernutrition was independently associated with overall TB recurrence (aHR 2.2, 95% CI: 1.1–4.1, *P* = 0.02) and with symptomatic TB recurrence (aHR 2.3, 95% CI: 1.1–4.5, *P* = 0.02), and showed elevated HR for asymptomatic TB recurrence (aHR 2.2, 95% CI: 0.9–6.9, *P* = 0.12), though it was not found to be statistically significant (Supplementary Data Tables S7–S9). Additionally, a positive month 2 culture was independently associated with asymptomatic TB recurrence in this extended cohort (aHR 2.7, 95% CI: 1.0–7.4, *P* = 0.06) (Supplementary Data Table S9).

## DISCUSSION

Our analysis showed that 8.4% of those with TB cure developed TB recurrence, which was often asymptomatic at the time of diagnosis. In our study, active screening for recurrent TB was performed every 6 months, so the high number of diagnoses made at or before the first screening visit suggests that earlier or more frequent screening may be beneficial, warranting further study. We found that younger individuals (age 14–35 years) were more likely (though not statistically significant) to have a TB recurrence diagnosed at an asymptomatic stage through scheduled microbiological testing. Undernutrition was associated with a two-fold higher risk of any TB recurrence diagnoses. To our knowledge, this is the first study to measure the burden of asymptomatic recurrent TB relative to that of symptom-driven diagnoses in India, the world’s largest TB epidemic that is not driven by HIV.

Among the few other studies, a South African study followed up PLWH quarterly for 3 years after TB treatment and found the cumulative incidence of asymptomatic TB to be 0.9 (0.5–1.4) per 100 PY. By comparison, our cohort with very few PLWH found a much higher incidence rate of 3.0 (IQR 1.8–4.6) per 100 PY, despite less frequent microbiological testing.^22^ One potential reason for this is that asymptomatic TB may progress more rapidly to symptomatic TB among PLWH compared to the HIV uninfected population.^26–28^

The majority of TB recurrence occurs soon after TB treatment.^15,29^ We also found that recurrence occurred within 10 months of treatment completion, and 50% of all recurrences were found within 6 months of treatment completion. Notably, of the recurrent TB that was diagnosed at scheduled study visits where microbiological testing was performed (6 or 12 months after treatment completion), the majority was asymptomatic. This suggests that a subset of people remain with asymptomatic TB for many months without progressing to symptomatic TB; this may be especially common among younger adults. Similarly, individuals diagnosed with symptomatic recurrent TB often lacked recent microbiological testing and may also have experienced extended asymptomatic periods prior to symptom onset. Furthermore, since undernutrition is a consistent risk factor for TB recurrence, those with undernutrition at treatment initiation and completion should be targeted for screening for TB recurrence.^30,31^

Our analysis has several limitations. First, the microbiological testing for recurrent TB was limited to one or two time points per participant up to 1 year after treatment completion. Earlier and more frequent testing would likely have identified more cases at an asymptomatic stage. Second, we used proportional hazards regression method for the risk factor analysis, though diagnosis of asymptomatic recurrence could occur only at certain time points. Therefore, the timing of diagnoses of recurrences reported above does not necessarily reflect the time to initial occurrence of the asymptomatic TB recurrence. Third, our cohort had a relatively small number of asymptomatic recurrence diagnoses. Fourth, longer-term outcomes were unavailable, as participants diagnosed with recurrent TB were not followed up further and were initiated on TB retreatment, limiting assessment of how often asymptomatic disease would have resolved without treatment.

## CONCLUSION

Our study provides much needed epidemiologic data on the hidden epidemic of asymptomatic TB recurrence. This may contribute to substantial TB transmission in communities in high-TB-burden countries. Further research should focus on the frequency and method of screening for asymptomatic TB recurrence, cost effective diagnostics, appropriate management strategies, and the natural progression of asymptomatic TB. Importantly, the contribution of asymptomatic TB mitigation to achieving 2030 TB elimination goals should be explored as it may generate much-needed political will in countries with a high TB burden.

## Supplementary Material
