# Supplementary material for: Asymptomatic and symptomatic recurrence of TB among cured pulmonary TB patients
Source: IJTLD Open. 2026 Oct 12;3(10):653–61. doi: 10.5588/ijtldopen.26.0210 (PMC13657022; doi:10.5588/ijtldopen.26.0210)
Supplement: Supplementary file 1 [file ijtldopen26-0210_supplementarydata1.pdf]

ONLINE DATA SUPPLEMENT

Supplementary figure 1: Forest plot showing risk factors for culture confirmed symptomatic tuberculosis (TB) recurrence among culture confirmed pulmonary TB cases with culture confirmed TB cure (N=713)

Characteristics	Symptomatic Recurrence n = 40; n(%)	Univariate HR (95% CI) (p-value)	Univariate 95 % CI	Multivariate HR (95% CI) (p-value)	Multivariate 95 % CI
**Sex**					
Male	29 (6.7%)	Ref		Ref	
Female	11 (4.3%)	0.7 (0.3 – 1.3) (0.23)		0.7 (0.3 – 1.5) (0.32)	
**Age (Years)**					
>35	24 (7.5%)	Ref		Ref	
14-35	16 (4.3%)	0.6 (0.3 – 1.2) (0.14)		0.4 (0.2 – 0.8) (0.01)	
**Body Mass Index**					
Normal	11 (4.2%)	Ref		Ref	
Obese	0 (0%)	..			
Underweight	29 (7.4%)	1.8 (0.9 – 3.8) (0.09)		2.2 (1.0 – 4.7) (0.04)	
**HIV Infection**					
No	39 (5.7%)	Ref			
Yes	1 (12.5%)	1.7 (0.1 – 7.8) (0.61)			
**Diabetes mellitus**					
No	26 (5.6%)	Ref			
Yes	14 (6.2%)	1.1 (0.6 – 2.0) (0.81)			
**Smoking**					
No	27 (5.4%)	Ref			
Yes	9 (7.1%)	1.3 (0.6 – 2.6) (0.53)			
**Alcohol Consumption**					
No	22 (4.9%)	Ref			
Yes	16 (7.3%)	1.3 (0.7 – 2.4) (0.46)			
**Treatment Regime**					
Thrice Weekly	35 (6.1%)	1.5 (0.7 – 4.4) (0.38)			
Daily	5 (4.1%)	Ref			
**Baseline AFB Smear**					
Negative	5 (4.1%)	Ref			
Positive	35 (6.1%)	1.5 (0.6 – 4.2) (0.43)			
**Month 2 AFB Smear**					
Negative	21 (4.2%)	Ref			
Positive	4 (7.3%)	1.5 (0.5 – 4.1) (0.43)			
**Month 2 Culture**					
Negative	28 (5.3%)	Ref			
Positive	7 (6.7%)	1.2 (0.5 – 2.6) (0.69)			

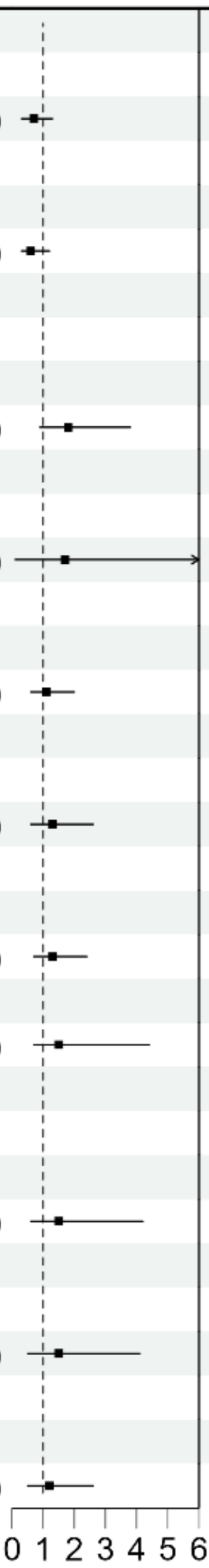

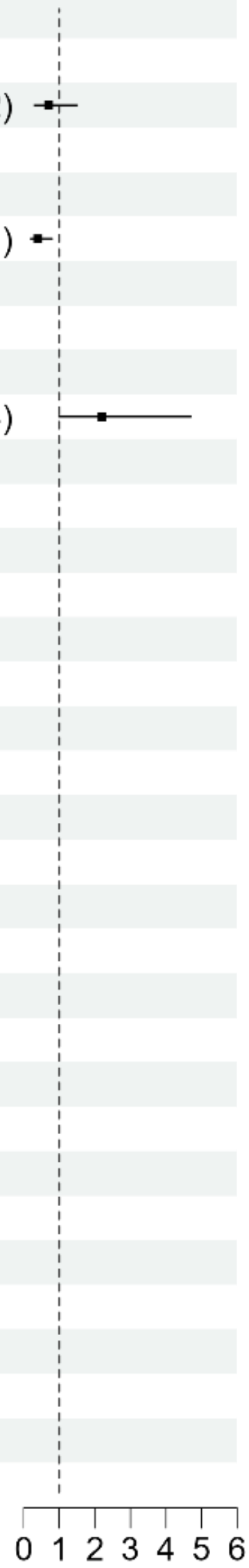

Supplementary table 1: Modes and timing of diagnosis of symptomatic and asymptomatic, confirmed tuberculosis recurrences, among adult culture confirmed pulmonary tuberculosis cases with culture confirmed cure (N = 713)

	**Symptomatic**	**Asymptomatic**
**Total number of diagnoses**	40	20
**Mode of diagnosis**		
Scheduled study visit	13	22
Unscheduled study visit	23	0
Outside of study	4	0
**Symptom duration at diagnosis, days**	18 (10-45)	NA
**Diagnoses at 18-month study visit**	1	11
**Diagnoses at 12-month study visit, TBDM study**	7	10

Supplementary table 2: Risk factors for confirmed tuberculosis recurrence (symptomatic or asymptomatic), among adult culture confirmed pulmonary tuberculosis cases with culture confirmed cure (N = 713)

Risk factors	N	TB recurrence, n (%)	Unadjusted Hazard Ratio uHR (95% CI), (p value)	Adjusted Hazard Ratio aHR (95% CI), (p value)
**Gender**				
Male	452	45 (10·0%)	Ref	Ref
Female	261	15 (5·7%)	0·6 (0·3 – 1·0) (0·07)	0·6 (0·3 – 1·2) (0·16)
**Age (Years)**				
> 35	323	29 (9·0%)	Ref	Ref
14 - 35	390	31 (7·9%)	1·0 (0·6 – 1·6) (0·94)	0·7 (0·4 – 1·2) (0·20)
**Body mass index**				
Normal	269	15 (5·6%)	Ref	Ref
Obese	38	1 (2·6%)	0.4 (0.0 – 2.1) (0.42)	0·5 (0.1 – 3·7) (0.48)
Underweight	405	44 (10·9%)	2·0 (1·1 – 3·7) (0·02)	2·1 (1·1 – 4·0) (0·03)
**HIV infection**				
No	701	59 (8·4%)	Ref	··
Yes	8	1 (12·5%)	1·1 (0·1 – 5·2) (0·90)	
**Diabetes mellitus**				
No	484	44 (9·1%)	Ref	··
Yes	229	16 (7·0%)	0·7 (0·4 – 1·3) (0·31)	
**Smoking**				
No	519	43 (8·3%)	Ref	··
Yes	128	11 (8·6%)	1·0 (0·5 – 1·9) (0·97)	
**Alcohol consumption**				
No	463	36 (7·8%)	Ref	··
Yes	223	21 (9·4%)	1·0 (0·6 – 1·8) (0·91)	
**Treatment Regime**				
Thrice Weekly	587	51 (8·7%)	1·2 (0·6 – 2·7) (0·56)	··
Daily	126	9 (7·1%)	Ref	
**Baseline AFB Smear**				
Negative	123	7 (5·7%)	Ref	··
Positive	590	53 (9·0%)	1·6 (0·8 – 3·8) (0·27)	
**Month 2 AFB Smear**				
Negative	514	35 (6·8%)	Ref	··
Positive	56	5 (8·9%)	1·2 (0·4 – 2.7) (0·74)	
**Month 2 Culture**				
Negative	541	38 (7·0%)	Ref	Ref
Positive	109	12 (11·0%)	1·5 (0·7 – 2·7) (0·25)	1·3 (0·7 – 2·5) (0.42)

* AFB- Acid fast bacilli

Supplementary table 3: Risk factors for culture confirmed tuberculosis recurrence (symptomatic), among adult culture confirmed pulmonary tuberculosis cases with culture confirmed cure (N = 693)

Risk factors	N	Clinical TB recurrence, n (%)	Unadjusted Hazard Ratio uHR (95% CI), (p value)	Adjusted Hazard Ratio aHR (95% CI), (p value)
**Gender**				
Male	436	29 (6·7%)	Ref	Ref
Female	257	11 (4·3%)	0·7 (0·3 – 1·3) (0·23)	0·7 (0·3 – 1·5) (0·32)
**Age (Years)**				
> 35	318	24 (7·5%)	Ref	Ref
14 - 35	375	16 (4·3%)	0·6 (0·3 – 1·2) (0·14)	0·4 (0·2 – 0·8) (0·01)
**Body mass index**				
Normal	265	11 (4·2%)	Ref	Ref
Obese	37	0 (0%)	··	··
Underweight	390	29 (7·4%)	1·8 (0·9 – 3·8) (0·09)	2·2 (1·0 – 4·7) (0·04)
**HIV infection**				
No	681	39 (5·7%)	Ref	··
Yes	8	1 (12·5%)	1·7 (0·1 – 7·8) (0·61)	
**Diabetes mellitus**				
No	466	26 (5·6%)	Ref	··
Yes	227	14 (6·2%)	1·1 (0·6 – 2·0) (0·81)	
**Smoking**				
No	503	27 (5·4%)	Ref	··
Yes	126	9 (7·1%)	1·3 (0·6 – 2·6) (0·53)	
**Alcohol consumption**				
No	449	22 (4·9%)	Ref	··
Yes	218	16 (7·3%)	1·3 (0·7 – 2·4) (0·46)	
**Treatment Regime**				
Thrice Weekly	571	35 (6·1%)	1·5 (0·7 – 4·4) (0·38)	··
Daily	122	5 (4·1%)	Ref	
**Baseline AFB Smear**				
Negative	121	5 (4·1%)	Ref	··
Positive	572	35 (6·1%)	1·5 (0·6 – 4·2) (0·43)	
**Month 2 AFB Smear**				
Negative	500	21 (4·2%)	Ref	··
Positive	55	4 (7·3%)	1·5 (0·5 – 4·1) (0·43)	
**Month 2 Culture**				
Negative	531	28 (5·3%)	Ref	··
Positive	104	7 (6·7%)	1·2 (0·5 – 2·6) (0·69)	

* AFB- Acid fast bacilli

Supplementary table 4: Risk factors for culture confirmed tuberculosis recurrence (asymptomatic), among adult culture confirmed pulmonary tuberculosis cases with culture confirmed cure (N = 673)

Risk factors	N	Subclinical TB recurrence, n (%)	Unadjusted Hazard Ratio uHR (95% CI), (p value)	Adjusted Hazard Ratio aHR (95% CI), (p value)
**Gender**				
Male	423	16 (3·8%)	Ref	Ref
Female	250	4 (1·6%)	0·4 (0·1 – 1·2) (0·13)	0·5 (0·1 – 1·5) (0·21)
**Age (Years)**				
> 35	299	5 (1·7%)	Ref	Ref
14 – 35	374	15 (4·0%)	2·7 (1·1 – 8·3) (0·05)	2·8 (0·8 – 9·8) (0·10)
**Body mass index**				
Normal	258	4 (1·6%)	Ref	Ref
Obese	38	1 (2.6%)	1.6 (0.1 – 10·8) (0·68)	3.2 (0.3 – 31.7) (0.33)
Underweight	376	15 (4·0%)	2.6 (0·9 – 9·1) (0·09)	2·0 (0·5 – 7·3) (0·30)
**HIV infection**				
No	662	20 (3·0%)	··	··
Yes	7	0 (0%)		
**Diabetes mellitus**				
No	458	18 (3·9%)	Ref	··
Yes	215	2 (0·9%)	0·2 (0·0 – 0·8) (0·05)	
**Smoking**				
No	492	16 (3·3%)	Ref	··
Yes	119	2 (1·7%)	0·5 (0·1 – 1·7) (0·33)	
**Alcohol consumption**				
No	441	14 (3·2%)	Ref	··
Yes	207	5 (2·4%)	0·6 (0·2 – 1·7) (0·39)	
**Treatment Regimen**				
Thrice Weekly	552	16 (2·9%)	0·9 (0·3 – 3·1) (0·83)	··
Daily	121	4 (3·3%)	Ref	
**Baseline AFB* Smear**				
Negative	118	2 (1·7%)	Ref	··
Positive	555	18 (3·2%)	1.9 (0·5 – 11·7) (0·41)	
**Month 2 AFB* Smear**				
Negative	493	14 (2·8%)	Ref	··
Positive	52	1 (1·9%)	0·6 (0·0 – 2·9) (0·61)	
**Month 2 Culture**				
Negative	513	10 (1·9%)	Ref	Ref
Positive	102	5 (4·9%)	2·3 (0·7 – 6·5) (0·12)	2·3 (0·8 – 6·8) (0·13)

* AFB- Acid fast bacilli

Supplementary table 5: Intensive phase pharmacokinetic drug concentrations and culture confirmed tuberculosis recurrence, among adult culture confirmed pulmonary tuberculosis cases with culture confirmed cure (N=713)

Anti-TB drug concentrations (µg/mL)	No recurrence n (row %)	TB recurrence n (row %)	Unadjusted Hazard Ratio uHR (95% CI), (p value)
**ASYMPTOMATIC TB RECURRENCE**			
**Rifampicin**			
Subtherapeutic (< 8)	267 (97)	9 (3)	-
Adequate (≥ 8)	44 (100)	0 (0)	
**Isoniazid**			
Subtherapeutic (< 3)	65 (97)	2 (3)	1·3 (0·3 – 6.3) (0·77)
Adequate (≥ 3)	262 (98)	6 (2)	Ref
**Pyrazinamide**			
Subtherapeutic (< 20)	56 (97)	2 (3)	1·9 (0·4 – 9·5) (0·44)
Adequate (≥ 20)	257 (98)	6 (2)	Ref
**SYMPTOMATIC TB RECURRENCE**			
**Rifampicin**			
Subtherapeutic (< 8)	267 (95)	15 (5)	0·8 (0·2 – 2·9) (0·79)
Adequate (≥ 8)	44 (94)	3 (6)	Ref
**Isoniazid**			
Subtherapeutic (< 3)	65 (96)	3 (4)	0·8 (0·2 – 2·7) (0·71)
Adequate (≥ 3)	262 (95)	15 (5)	Ref
**Pyrazinamide**			
Subtherapeutic (< 20)	56 (93)	4 (7)	1·5 (0·5 – 4·5) (0·50)
Adequate (≥ 20)	257 (95)	13 (5)	Ref
**TB RECURRENCE**			
**Rifampicin**			
Subtherapeutic (< 8)	267 (92)	24 (8)	1·3 (0·4 – 4·5) (0·63)
Adequate (≥ 8)	44 (94)	3 (6)	Ref
**Isoniazid**			
Subtherapeutic (< 3)	65 (93)	5 (7)	0·9 (0·3 – 2·4) (0·87)
Adequate (≥ 3)	262 (93)	21 (7)	Ref
**Pyrazinamide**			
Subtherapeutic (< 20)	56 (90)	6 (10)	1·6 (0·6 – 3·9) (0·33)
Adequate (≥ 20)	257 (93)	19 (7)	Ref

Supplementary table 6: Intensive phase pharmacokinetic drug concentrations and culture confirmed tuberculosis recurrence, among culture confirmed pulmonary tuberculosis cases with clinical response with or without culture confirmed cure (N= 752)

Anti-TB drug concentrations (µg/mL)	No recurrence n (row %)	TB recurrence n (row %)	Unadjusted Hazard Ratio uHR (95% CI), (p value)
**ASYMPTOMATIC TB RECURRENCE**			
**Rifampicin**			
Subtherapeutic (< 8)	269 (97)	9 (3)	-
Adequate (≥ 8)	46 (100)	0 (0)	
**Isoniazid**			
Subtherapeutic (< 3)	67 (97)	2 (3)	1·3 (0·3 – 6.2) (0·78)
Adequate (≥ 3)	266 (98)	6 (2)	Ref
**Pyrazinamide**			
Subtherapeutic (< 20)	56 (97)	2 (3)	1·9 (0·4 – 9·7) (0·42)
Adequate (≥ 20)	262 (98)	6 (2)	Ref
**SYMPTOMATIC TB RECURRENCE**			
**Rifampicin**			
Subtherapeutic (< 8)	269 (94)	16 (6)	0·9 (0·3 – 3·2) (0·91)
Adequate (≥ 8)	46 (94)	3 (6)	Ref
**Isoniazid**			
Subtherapeutic (< 3)	67 (96)	3 (4)	0·7 (0·2 – 2·5) (0·62)
Adequate (≥ 3)	266 (94)	17 (6)	Ref
**Pyrazinamide**			
Subtherapeutic (< 20)	56 (92)	5 (8)	1·9 (0·7 – 5·2) (0·24)
Adequate (≥ 20)	262 (95)	13 (5)	Ref
**TB RECURRENCE**			
**Rifampicin**			
Subtherapeutic (< 8)	269 (94)	25 (6)	1·5 (0·4 – 4·8) (0·54)
Adequate (≥ 8)	46 (94)	3 (6)	Ref
**Isoniazid**			
Subtherapeutic (< 3)	67 (96)	5 (4)	0·9 (0·3 – 2·3) (0·79)
Adequate (≥ 3)	266 (94)	22 (6)	Ref
**Pyrazinamide**			
Subtherapeutic (< 20)	56 (92)	7 (8)	1·8 (0·8 – 4·4) (0·17)
Adequate (≥ 20)	262 (95)	19 (5)	Ref

Supplementary table 7: Risk factors for culture confirmed tuberculosis recurrence (asymptomatic and symptomatic), among culture confirmed pulmonary tuberculosis cases with clinical response with or without culture confirmed cure (N= 752)

Risk factors	N	TB recurrence, n (%)	Unadjusted Hazard Ratio uHR (95% CI), (p value)	Adjusted Hazard Ratio aHR (95% CI), (p value)
**Gender**				
Male	478	50 (10·5%)	Ref	Ref
Female	274	15 (5·5%)	0·5 (0·3 – 0·9) (0·03)	0·6 (0·3 – 1·1) (0·08)
**Age (Years)**				
> 35	340	30 (8·8%)	Ref	Ref
14 - 35	412	35 (8·5%)	1·1 (0·7 – 1·7) (0·80)	0·7 (0·4 – 1·3) (0·30)
**Body mass index**				
Normal	282	16 (5·7%)	Ref	Ref
Obese	42	2 (4·8%)	0.8 (0.1 – 2.8) (0.77)	1.0 (0.2 – 4.4) (>0.99)
Underweight	427	47 (11·0%)	2·0 (1·2 – 3·7) (0·02)	2·2 (1·1 – 4·1) (0·02)
**HIV infection**				
No	739	64 (8·7%)	Ref	··
Yes	9	1 (11·1%)	1·0 (0·1 – 4·4) (0·98)	
**Diabetes mellitus**				
No	505	48 (9·5%)	Ref	··
Yes	247	17 (6·9%)	0·7 (0·4 – 1·2) (0·21)	
**Smoking**				
No	544	46 (8·5%)	Ref	··
Yes	141	13 (9·2%)	1·1 (0·6 – 1·9) (0·85)	
**Alcohol consumption**				
No	488	36 (7·4%)	Ref	··
Yes	236	26 (11·0%)	1·3 (0·8 – 2·1) (0·32)	
**Treatment Regime**				
Thrice Weekly	618	56 (9·1%)	1·4 (0·7 – 3·0) (0·39)	··
Daily	134	9 (6·7%)	Ref	
**Baseline AFB Smear**				
Negative	133	9 (6·8%)	Ref	··
Positive	619	56 (9·0%)	1·3 (0·7 – 2·8) (0·45)	
**Month 2 AFB Smear**				
Negative	538	39 (7·2%)	Ref	··
Positive	59	5 (8·5%)	1.0 (0·4 – 2.4) (0·96)	
**Month 2 Culture**				
Negative	558	40 (7.2%)	Ref	Ref
Positive	114	13 (11.4%)	1.5 (0.8 – 2.7) (0.21)	1.4 9 (0.7 – 2.5) (0.35)

* AFB- Acid fast bacilli

Supplementary table 8: Risk factors for culture confirmed tuberculosis recurrence (symptomatic), among culture confirmed pulmonary tuberculosis cases with clinical response with or without culture confirmed cure (N= 730)

Risk factors	N	Symptomatic TB recurrence, n (%)	Unadjusted Hazard Ratio uHR (95% CI), (p value)	Adjusted Hazard Ratio aHR (95% CI), (p value)
**Gender**				
Male	460	32 (7·0%)	Ref	Ref
Female	270	11 (4·1%)	0·6 (0·3 – 1·1) (0·13)	0·6 (0·3 – 1·3) (0·23)
**Age (Years)**				
> 35	335	25 (7·5%)	Ref	Ref
14 - 35	395	18 (4·6%)	0·7 (0·4 – 1·2) (0·19)	0·6 (0·3 – 1·1) (0·08)
**Body mass index**				
Normal	277	11 (4·0%)	Ref	Ref
Obese	41	1 (2·4%)	0.6 (0.0 – 3.0) (0.61)	0.6 (0.1 – 4.3) (0.57)
Underweight	411	31 (7·5%)	2·0 (1·0 – 4·1) (0·05)	2·3 (1·1 – 4·5) (0·02)
**HIV infection**				
No	717	42 (5·9%)	Ref	..
Yes	9	1 (11·1%)	1·5 (0·1 – 6·7) (0·71)	
**Diabetes mellitus**				
No	485	28 (5·8%)	Ref	..
Yes	245	15 (6·1%)	1·0 (0·5 – 1·9) (0·89)	
**Smoking**				
No	527	29 (5·5%)	Ref	..
Yes	138	10 (7·2%)	1·3 (0·6 – 2·5) (0·50)	
**Alcohol consumption**				
No	474	22 (4·6%)	Ref	..
Yes	229	19 (8·3%)	1·5 (0·8 – 2·8) (0·17)	
**Treatment Regime**				
Thrice Weekly	600	38 (6·3%)	1·7 (0·7 – 4·9) (0·28)	..
Daily	130	5 (3·8%)	Ref	
**Baseline AFB Smear**				
Negative	131	7 (5·3%)	Ref	..
Positive	599	36 (6·2%)	1·1 (0·5 – 2·7) (0·82)	
**Month 2 AFB Smear**				
Negative	523	24 (4·6%)	Ref	..
Positive	58	4 (6·9%)	1.3 (0·4 – 3.4) (0·61)	
**Month 2 Culture**				
Negative	548	30 (5.5%)	Ref	
Positive	108	7 (6.5%)	1.1 (0.4 – 2.3) (0.84)	

* AFB- Acid fast bacilli

Supplementary table 9: Risk factors for culture confirmed tuberculosis recurrence (asymptomatic), among culture confirmed pulmonary tuberculosis cases with clinical response with or without culture confirmed cure (N = 709)

Risk factors	N	Asymptomatic TB recurrence, n (%)	Unadjusted Hazard Ratio uHR (95% CI), (p value)	Adjusted Hazard Ratio aHR (95% CI), (p value)
**Gender**				
Male	446	18 (4·0%)	Ref	Ref
Female	263	4 (1·5%)	0·4 (0·1 – 1·0) (0·08)	0·4 (0·1 – 1·4) (0·15)
**Age (Years)**				
> 35	315	5 (1·6%)	Ref	Ref
14 - 35	394	17 (4·3%)	3·1 (1·2 – 9·3) (0·03)	3·1 (0·9 – 10·4) (0·07)
**Body mass index**				
Normal	271	5 (1·8%)	Ref	Ref
Obese	41	1 (2.4%)	1.3 (0.1 – 7.9) (0.82)	3.5 (0.3 – 35.3) (0.29)
Underweight	396	16 (4·0%)	2.2 (0·9 – 6·9) (0·12)	2·2 (0·6 – 7·9) (0·24)
**HIV infection**				
No	697	22 (3·2%)	··	··
Yes	8	0 (0%)		
**Diabetes mellitus**				
No	477	20 (4·2%)	Ref	··
Yes	232	2 (0·9%)	0·2 (0·0 – 0·7) (0·03)	
**Smoking**				
No	515	17 (3·3%)	Ref	··
Yes	131	3 (2·3%)	0·7 (0·2 – 2·0) (0·51)	
**Alcohol consumption**				
No	466	14 (3·0%)	Ref	··
Yes	217	7 (3·2%)	0.9 (0·3 – 2·2) (0·83)	
**Treatment Regimen**				
Thrice Weekly	580	18 (3·1%)	1.0 (0·4 – 3·4) (0·99)	··
Daily	129	4 (3·1%)	Ref	
**Baseline AFB* Smear**				
Negative	126	2 (1·6%)	Ref	··
Positive	583	20 (3·4%)	2.1 (0·6 – 13·2) (0·32)	
**Month 2 AFB* Smear**				
Negative	514	15 (2·9%)	Ref	··
Positive	55	1 (1·8%)	0·5 (0·0 – 2·6) (0·54)	
**Month 2 Culture**				
Negative	528	10 (1.9%)	Ref	Ref
Positive	107	6 (5.6%)	2.7 (0.9 – 7.4) (0.05)	2.7 (1.0 – 7.4) (0.06)

* AFB- Acid fast bacilli
